# A bi-directional segmentation method for prostate ultrasound images under semantic constraints

**DOI:** 10.1038/s41598-024-61238-5

**Published:** 2024-05-22

**Authors:** Zexiang Li, Wei Du, Yongtao Shi, Wei Li, Chao Gao

**Affiliations:** 1 College of Electrical Engineering and New Energy, China Three Gorges University, Yichang, Hubei, 443002 China; 2 College of Computer and Information Technology, China Three Gorges University, Yichang, Hubei, 443002 China; 3 Hubei Key Laboratory of Intelligent Vision Monitoring for Hydropower Engineering, China Three Gorges University, Yichang, Hubei, 443002 China

**Keywords:** Computational models, Data processing, Image processing

## Abstract

Due to the lack of sufficient labeled data for the prostate and the extensive and complex semantic information in ultrasound images, accurately and quickly segmenting the prostate in transrectal ultrasound (TRUS) images remains a challenging task. In this context, this paper proposes a solution for TRUS image segmentation using an end-to-end bidirectional semantic constraint method, namely the BiSeC model. The experimental results show that compared with classic or popular deep learning methods, this method has better segmentation performance, with the Dice Similarity Coefficient (DSC) of 96.74% and the Intersection over Union (IoU) of 93.71%. Our model achieves a good balance between actual boundaries and noise areas, reducing costs while ensuring the accuracy and speed of segmentation.

## Introduction

According to statistical data^1^, about half of all men are prone to prostatitis, while approximately 30–40% of men aged 25–40 have chronic prostatitis to varying degrees. Prostate cancer is a prevalent malignancy among men, treated primarily using androgen deprivation therapy (ADT)^2^ in cases of intermediate and high-risk prostate cancer. However, most patients will progress to castration-resistant prostate cancer (CRPC) within 1.5–2 years of ADT, which is usually fatal^3^. To gather information about prostate pathology, medical research and practice frequently rely on measuring its shape, borders, and volume to aid physicians in making accurate diagnoses. Transrectal ultrasound (TRUS)^4^ is a widely used clinical method for prostate examination.

Currently, the TRUS imaging technology is plagued by problems such as low contrast, poor resolution, and interference from speckle noise due to the influence of imaging equipment, imaging principles, and individual differences. Furthermore, the accuracy of segmentation is affected by greyscale inhomogeneity, interference from echo loss, and artifacts arising from echo texture. Technically, the manual segmentation method is predominantly utilized in the medical domain, and it is not only resource-intensive but also produces inconsistent results due to variations in the segmentation styles of different physicians. Such a process has a significant impact on the segmentation outcomes^5^.

Compared with the manual segmentation method, automatic segmentation technologies are more efficient. Auto-segmentation techniques fall under two categories: traditional algorithms and deep learning algorithms. The rapid development of technology has allowed the two technologies to merge. This extends to the incorporation of various image features in the methods, such as the combination of semantic and pixel information. Consequently, we classify these algorithms into three groups: traditional segmentation methods, segmentation based on a learning model, and hybrid segmentation methods.

### Traditional segmentation methods

Among the traditional segmentation methods, Mumford and Shah^6^ solved the variability problem of level sets, making the Active Contour Modeling^7^ to the most widely used segmentation technology based on level sets. On this basis, Wang^8^ utilized a Gaussian probabilistic model for establishing statistical learning of previous shapes and a cosine function for energy term fitting. However, its robustness is not optimal. In addition, Hodge^9^ proposed a semi-automated algorithm to segment the boundaries of prostate ultrasound images. This algorithm is based on a 2D Active Shape Model (ASM) and 3D rotated slices, which utilize a point-distribution model for segmenting prostate boundaries. However, the accuracy of this segmentation method can be improved. Previous traditional methods have specific flaws in their initialization processes, such as manual positioning.

### Segmentation based on learning model

Due to the lack of adequate datasets for medical images, the designed framework should not be too deep. Lightweight neural network architecture aims to design network structures with lower computational complexity. In general, neural networks use encoding and decoding structures to handle segmentation tasks. The encoder extracts the contextual information while the decoder captures the segmentation areas. The classical U-net^10^ and Segnet^11^ perform well in small-scale data segmentation but the accuracy still needs to be improved in the ultrasound images. BisenetV2^12^ uses an aggregation layer that includes semantics and details to enhance feature representation.

Nonetheless, effective semantic information cannot be extracted due to image quality, and boundary recognition is more likely to be disturbed. Luana^13^ used the DeepLabv3 + 2.5D model with DPN-131 encoder to segment renal tumors from CT scans. Although target segmentation has a DSC of 85.17%, the performance is limited by the boundary of this encoder. BCRNN^14^ adopts polar coordinate processing from the perspective of prostate physiological features. Its optimal Dice coefficient is 92.39%, but when the boundary is far away from the center point and the polar angle is slight, the selected pixels tend to be duplicated, which results in a large amount of data redundancy. When the polar angle is large, there are gaps in the selected pixels, which results in information loss. The training data required for deep learning is vast. However, there is often little data available in medical imaging because it involves private information about patients, and the labeling of images increases the workload of doctors. In addition, deep learning has significant challenges in the TRUS datasets with small data volumes and complex images: the first point is that the large amount of noise on TRUS requires deeper neural networks and a large number of parameters to be trained, which undoubtedly adds a large amount of expense and time cost; the second point is that as the number of layers in the network or the number of training rounds increases, the degree of overfitting of the small-scale medical image data is more serious, which makes the model in the training set to perform better, but the performance on the test set decreases; the third point, fuzzy boundaries increase in the confidence region of the neural network, resulting in limited segmentation.

### Hybrid segmentation methods

A mixture of methods or features is a widely used hybrid segmentation method. Li^15^ used the SOLOv2 network and level sets to segment instances. To penalize the Active Contour, Suvidha^16^ used a modified U-Net architecture for learning penalization values. The proposed hybrid deep learning model fuses modern deep learning segmentation algorithms with traditional active contour segmentation techniques. Similarly, a new Revised Closed K-segment Principal Curve (RCKPC) method proposed by the H-ProSeg^17^ segmentation framework has a DSC of 95.8% through the network training parameters; lastly, the method obtains the initial optimal parameters (i.e., weights and thresholds) of the Adaptive Learning Rate Backpropagation Neural Network (ABPNN), a network that only learns a few coordinates to perform the shape restoration role. Although the RCKPC method can strictly match the morphological features of the prostate, it directly abandons pixel information in controversial regions, which will lead to severe errors. The PTN^18^ network processes prostate ultrasound images from the perspective of polar coordinate space, converts the pixel classification problem into a regression problem and obtains 89.97% DSC segmentation, which reduces the parameters by 18–41% compared with other convolutional networks but relies heavily on the determination of the center point. The hybrid approach is experimentally validated as a better segmentation method because this method combines the semantic information and pixel features of the image more effectively.

Note that hybrid segmentation methods overcome the limitations of traditional algorithms and also reduce the time and space complexity of deep learning algorithms. Based on this, we propose a bidirectional segmentation method that relies on semantic constraints to construct the boundary points of the prostate region. The primary contributions of this method include:

1. A semantic constraint is proposed to represent the ultrasound image, which restricts the region to be segmented to a specific area. This ensures uniform expression of pixel and shape features in the image. Additionally, a transform localized convolutional network is designed for initialized localization.
2. To mitigate the excessive number of outliers in the segmentation results caused by one or more values with considerably higher differences than the others, we will introduce neighbourhood information to the operator. Furthermore, we propose an exponential time series denoising algorithm to address the outliers detected within a small region.
3. The operator and denoising algorithm are combined for two-way iteration to detect and recover deviations caused by sudden changes in grey level differences. This improves outlier resolution in non-significant regions and brings the significant region closer to the actual boundary point.

## Method

**Figure 1:**
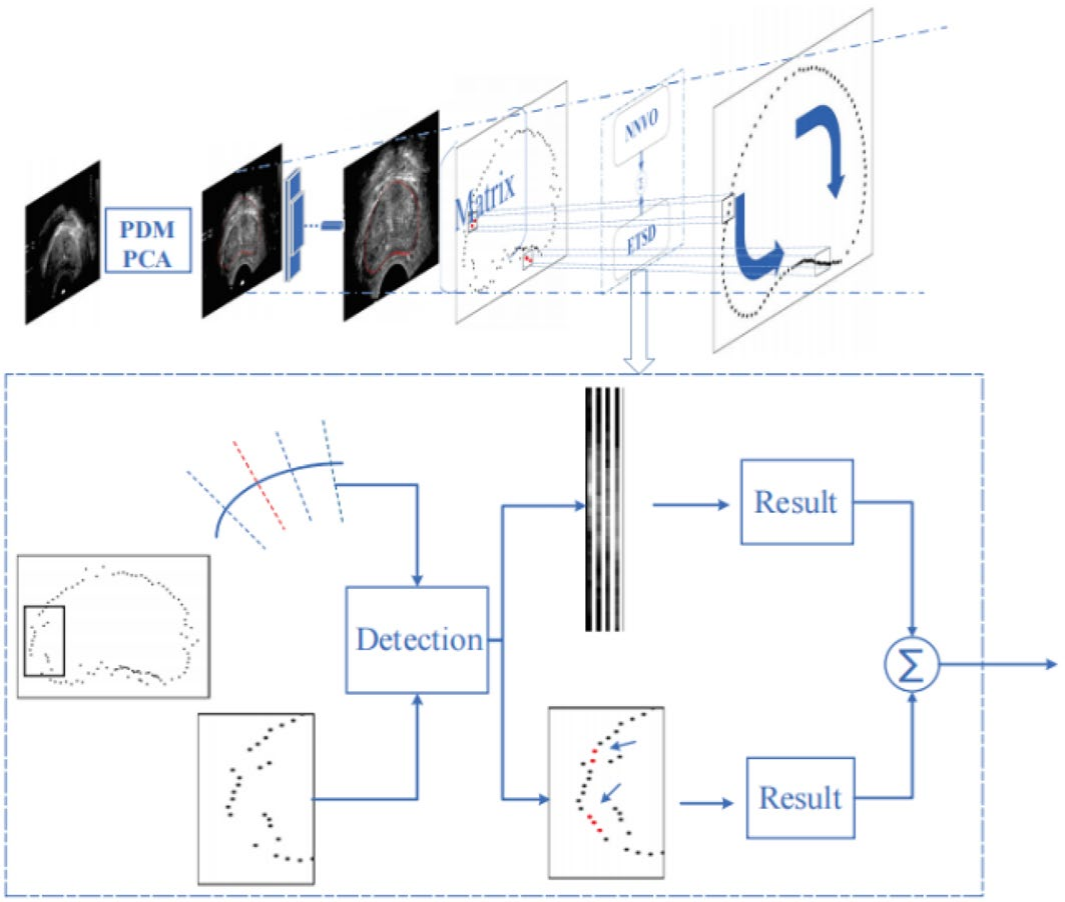
Flowchart of the BiSeC model (NNVO, Neighbourhood normal vector operator. ETSD, Exponential time series denoising).

Figure 1 shows the BiSeC model for prostate segmentation in TRUS images. It is a bidirectional segmentation method under semantic constraints that aims to improve segmentation accuracy in medical images. The technique combines web and traditional methods and uses a weighted fusion of grey and morphological features in a three-phase framework. In the first phase, the shape space is constructed using a point distribution model (PDM) and principal component analysis (PCA) to obtain the prior shapes. These shapes are then initially localized by the designed convolutional network. In the second phase, the semantic constraints matrix is obtained through the semantically constrained representation of ultrasound images. This helps to reduce the adverse effects of high noise. In the third phase, outliers in the results obtained from the Hodge method are detected using the semantic constraint expression. After detection, the final segmentation results are acquired through a weighted combination of the neighborhood average vector operator and exponential time series denoising.

### Model building and initial localization of convolutional networks

Using the whole image for data input, the algorithm is highly susceptible to the characteristics of ultrasound images, such as low resolution and severe noise, which leads to reduced segmentation accuracy. Therefore, it is necessary to improve this phenomenon by using particular prostate expression patterns. In this proposal, the pixel information of the image is extracted and sampled based on semantic constraints to reduce the unfavourable effects of noise speckles and combined with the shape information of the prostate for expression. The statistical model is constructed by first extracting and modeling the contour features of the image by PDM^19^ and PCA^20^; then, based on the model, the image is sampled to express the pixel information and contour information.

**Figure 2:**
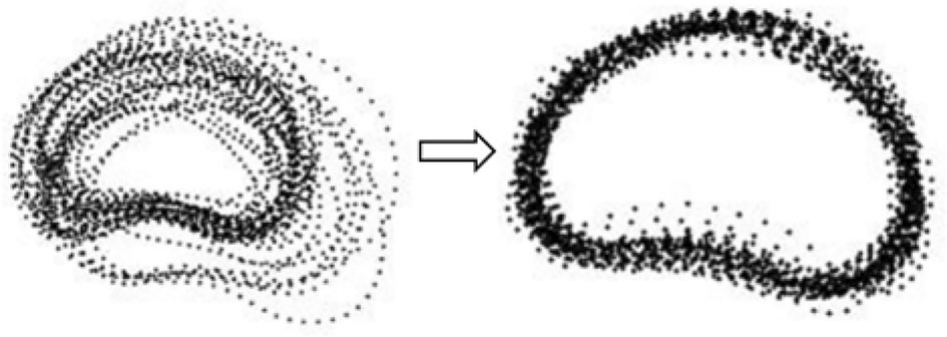
Diagram of PDM construction.

The construction's specific implementation process entails utilizing a shape description function. Firstly, the graph's boundaries are depicted by points, and the pertinent points are identified as landmarked points. Secondly, calibration is essential for comparing equivalent points of varying shapes, necessitating the normalization of all these points to a coordinate system. Procrustes methods are used for normalized calibration, including rotation, scaling, and transformation of the training shapes to bring the equivalent points of each shape closer together. A similarity transformation is used to bring each sample together as closely as possible. Its main objective is to minimize the sum of squares of distances, which are weighted. A fixed number of boundary contours are picked and applied with resampling procedures. Finally, the training set is standardized by transforming and projecting it into a unified coordinate system. Figure 2 is a schematic diagram of PDM.

(1) PDM constitutes a 2D variation space, assuming that the points are distributed in the allowable shape domain. The distribution shape is an ellipsoid in 2n space, and the shape model is obtained:

1 $${\overline{\text{x}}} = \frac{1}{{\text{n}}}\sum\limits_{{{\text{i}} = 1}}^{{\text{n}}} {{\text{x}}_{{{\text{i}}_{{}} }} }$$

(2) The axes were determined using PCA, with each axis generating a pattern of variation for which a covariance matrix was calculated:

2 $$C{\text{ov}} = \frac{1}{{\text{n}}}\sum\limits_{{{\text{i}} = 1}}^{{\text{n}}} {(x_{i} - \overline{x})} (x_{i} - \overline{x})^{T}$$

(3) Calculate all the eigenvalues $\lambda$ and eigenvectors of the covariance matrix *P*, with the first t chosen from largest to smallest to satisfy the following requirements:

3 $$\frac{{\sum\limits_{{{\text{i}} = 1}}^{t} {\lambda_{i} } }}{{\sum\limits_{s = 1}^{n} {\lambda_{s} } }} > \mu \sum \lambda$$

Among them, $\mu$ represents the proportion of the selected feature among all features, generally ranging from 95 to 98%;

(4) The new plan shape is decomposed enough to be represented by an average graph ${\overline{\text{x}}}$ and eigenvectors $P$:

4 $${\text{s}} = P^{T} (x - \overline{x})$$

In the training shape space, the approximated shape is a linear combination of feature vectors ${\hat{\text{x}}} = {\overline{\text{x}}} + {\text{Ps}}$, from which the center shape can be obtained.

**Figure 3:**
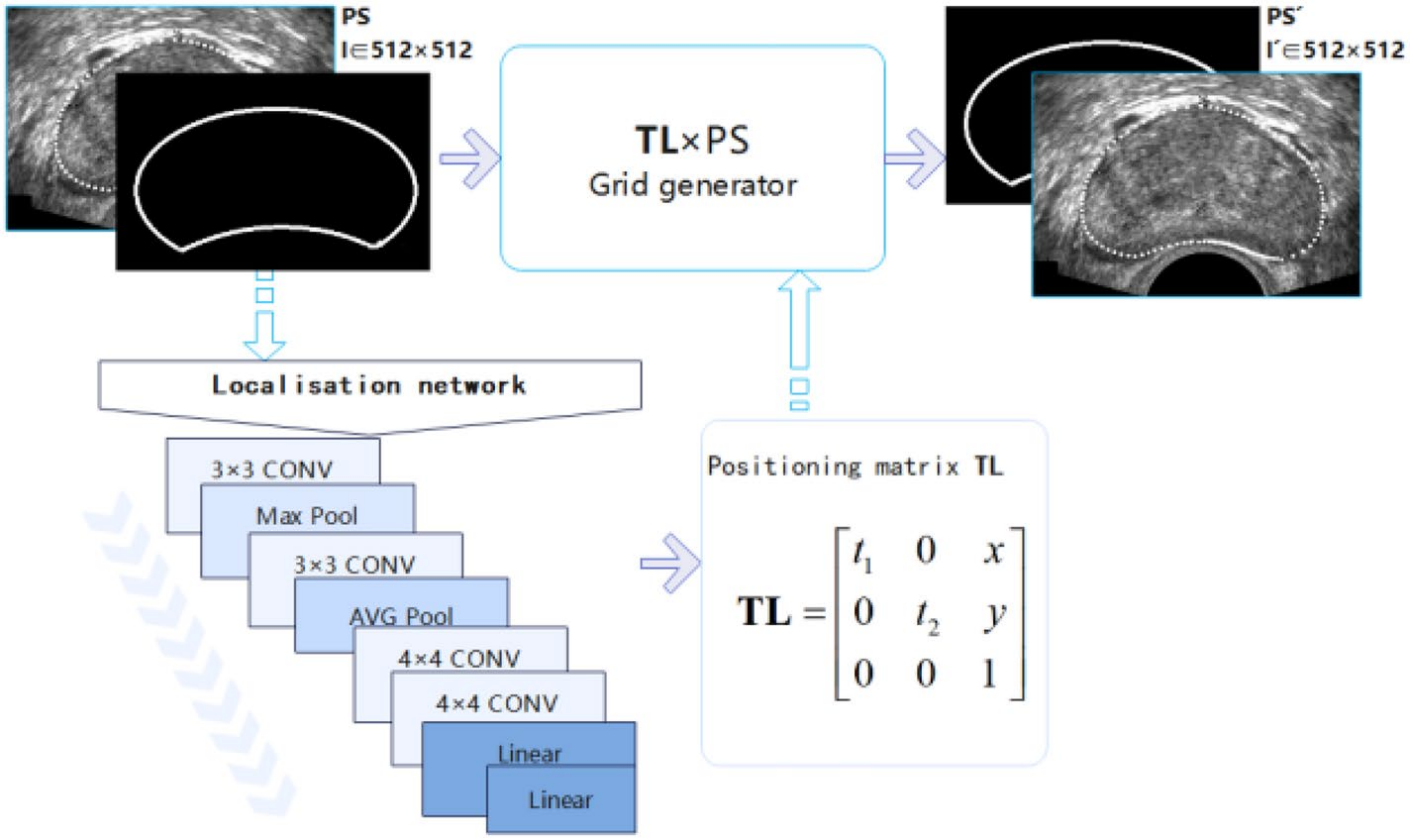
Convolutional network model for initial localization.

To solve the pain point that traditional segmentation methods can not be automatically localized, consider the disadvantages of neural networks, including high cost and low accuracy. The DeepPrint model^21^ in Joshua's method refers to the design of a transformed localization convolutional network for initialization. To realize this localization, the TRUS image and coordinate points are transformed into a 512 × 512 image with white outline on a black background; through the localization network, the parameters of the affine transformation are output, and the localization affine matrix is generated through the parameters, the transform localization matrix coefficients, including displacement coefficients1 $\left[ {{\text{x}},y} \right]$, scaling coefficients $\left[ {{\text{t}}_{{1}} ,{\text{t}}_{2} } \right]$ (t1 means scaling in the horizontal direction, and t2 means compression in the vertical direction) are obtained by the network, and the transform localization matrix is generated. The convolutional network model used for initial localization is shown in Fig. 3.

The TL output from the network may generate small numbers, and as a result, the coordinates obtained may also be small. However, the required coordinates are integers, and the use of standard rounding methods can lead to a situation where the gradient fails to descend when the parameters of the TL are changed on a small scale without a change in the coordinate parameters. Therefore, a Parameterized Sampling Grid is used, which uses a bilinear difference applied to the updated TL parameters, resulting in a microscopic mapping between the output image $I^{\prime}$ and the input image $I$.

5 $${\mathrm{I^{\prime}}}_{{\text{i}}}^{{\text{c}}}=\sum_{{\text{n}}}^{{\text{H}}} \sum_{{\text{m}}}^{{\text{W}}} {{\text{I}}}_{{\text{nm}}}^{{\text{c}}}{{\max}}\left(\mathrm{0,1}-\left|{{\text{x}}}_{{\text{i}}}-{\text{m}}\right|\right){{\max}}\left({0,1}-\left|{{\text{y}}}_{{\text{i}}}-{\text{n}}\right|\right)$$

where $I_{{{\text{nm}}}}^{{\text{c}}}$ is the pixel value of PS at (n, m), on channel c; $I_{{\text{i}}}^{{\prime {\text{c}}}}$ is the pixel value of *PS*′ at (x, y), on channel c. H and W are the height and width of the grid. The bilinear difference allows us to handle the occurrence of fractional coordinates and facilitates the gradient descent of the network to find the optimal solution.

**Figure 4:**
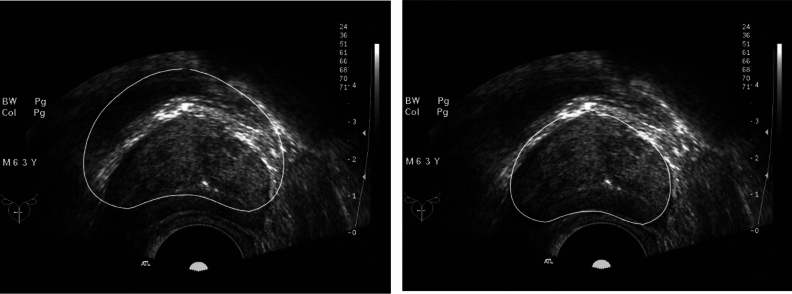
Schematic of localization results.

The positioning result is shown in Fig. 4. The transformed localization matrix TL is applied to the priori shape PS such that the initial localization, $PS = TL \times PS$. To satisfy the operation rule, PS needs to be filled with one dimension to obtain $PS_{3 \times N}$. Our group found that the effect of localization accuracy will be corrected in the subsequent model, i.e., the maximum error of each pixel on the average vector can be up to 2L (2L represents the length of the average vector) length confidence range and such a confidence range can hardly exist in the trained network, so to save the cost and time, the localization convolutional network was designed to consist mainly of four convolutional blocks and three fully connected layers. Instead of supervising the transformations through reference points (like cores), the underlying network and the representation extraction network tell the localization network what a "good" transformation is so that it can learn a more discriminative representation of the input prostate.

### Semantic constraint sampling

**Figure 5:**
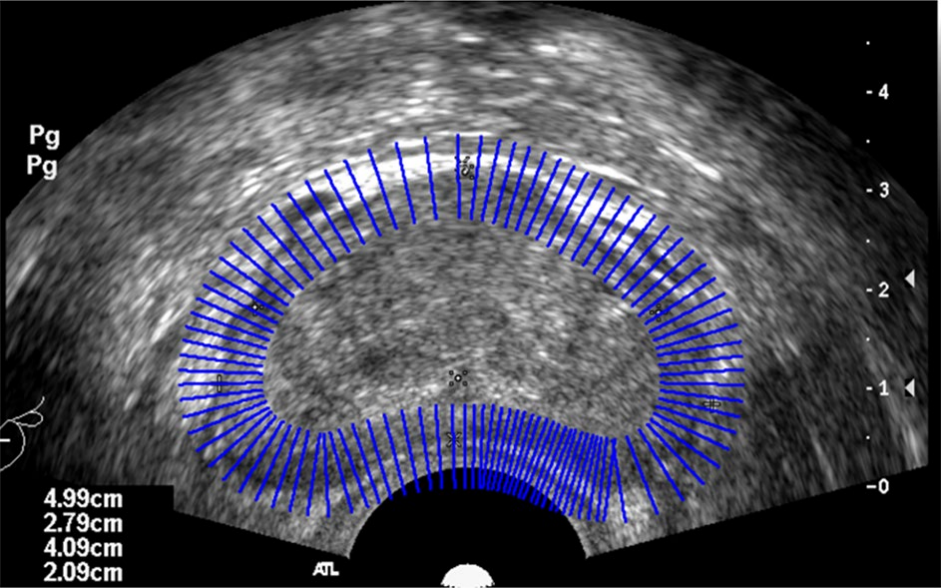
Schematic diagram of sampling point normal vector.

A priori shape PS is localized on the TRUS image to be segmented to form an approximate contour curve. Practice vectors are sampled at n points on the curve, with the prescribed method vector pointing out of the curve. A schematic diagram is shown in Fig. 5.

At the kth sampling point ${\rm idx}_{- \rm k}$ of the PS contour, the positive and negative directions of the normal vector of length L are taken. Each point has its corresponding row index value, gray scale value g, and a function $f\left( k \right) = [idx_{ - L,k} ,idx_{ - L + 1,k} \cdots idx_{1,k} ,idx_{2,k} \cdots idx_{L,k} ]$. Set the number of sampling points to N, and then have ${\text{idx}}_{{{\text{ - L}} \cdot {\text{L,k}}}}$, a $N \times 2L$ semantic constraint matrix $IM_{N \times 2L}$ is created by the function $f$. $IM$ represents the sampled normal vector index values.

6 $$IM_{N \times 2L} = [f(1),f(2),f(3), \cdots ,f(N)] = \left[ {\begin{array}{*{20}c} {idx_{ - L,1} } & {idx_{ - L + 1,1} } & \cdots & {idx_{ - 1,1} } \\ {idx_{ - L,2} } & {idx_{ - L + 1,2} } & \cdots & {idx_{ - 1,2} } \\ {idx_{ - L,3} } & {idx_{ - L + 1,3} } & \cdots & {idx_{ - 1,3} } \\ {\begin{array}{*{20}c} {} \\ {idx_{ - L,N} } \\ \end{array} } & {\begin{array}{*{20}c} {} \\ {idx_{ - L + 1,N} } \\ \end{array} } & {\begin{array}{*{20}c} \vdots \\ \cdots \\ \end{array} } & {\begin{array}{*{20}c} {} \\ {idx_{ - 1,N} } \\ \end{array} } \\ \end{array} \begin{array}{*{20}c} {idx_{1,1} } & {idx_{2,1} } & \cdots & {idx_{L,1} } \\ {idx_{1,2} } & {idx_{2,2} } & \cdots & {idx_{L,2} } \\ {idx_{1,3} } & {idx_{2,3} } & \cdots & {idx_{L,3} } \\ {\begin{array}{*{20}c} {} \\ {idx_{1,N} } \\ \end{array} } & {\begin{array}{*{20}c} {} \\ {idx_{2,N} } \\ \end{array} } & {\begin{array}{*{20}c} \vdots \\ \cdots \\ \end{array} } & {\begin{array}{*{20}c} {} \\ {idx_{L,N} } \\ \end{array} } \\ \end{array} } \right]$$

**Figure 6:**
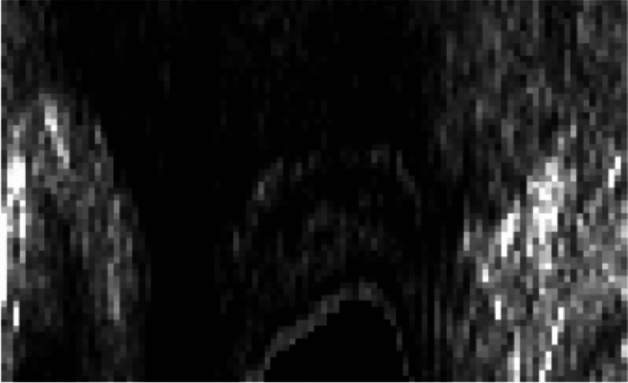
Vertical narrow band schematic diagram. ($GM_{100 \times 2L}^{T}$, consisting of 100 normal vectors).

Each index value has a corresponding grayscale value, so the grayscale matrix and the index value matrix form a bijection relationship, and an average vector grayscale matrix exists $GM_{N \times 2L} = \phi (IM_{N \times 2L} )$. This proposal establishes a semantic constraint matrix to express the whole ultrasound image to be segmented through semantic information. Subsequently, based on the constraint matrix, the image is segmented using a neighbourhood average vector operator and an exponential time series denoising algorithm. This expression reduces the interference of noise on the ultrasound image, reduces the size of the data and shortens the time consumption. For ease of observation, Fig. 6 shows a visualization of the set of narrow-band normal vectors, the matrix $GM_{N \times 2L}^{T}$, which is zoomed in by a factor of 15.

### Bidirectional hybrid segmentation

#### Neighbourhood normal vector operator

**Figure 7:**
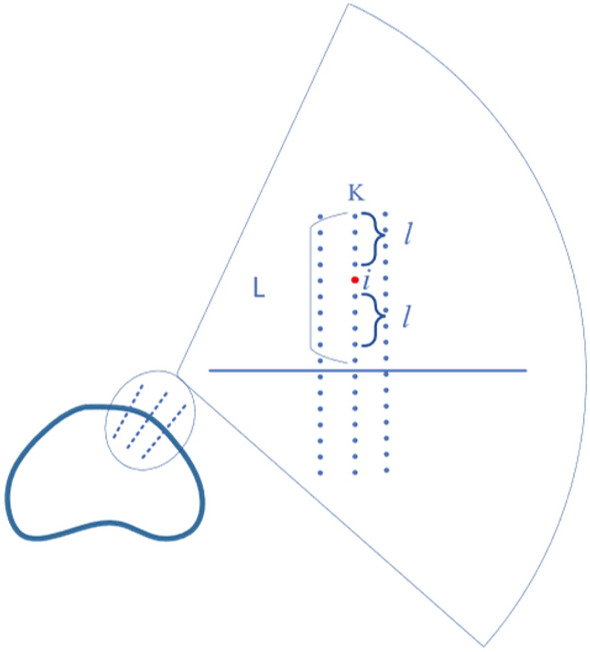
General vector search description diagram. Search within the upper and lower bounds of L, centered on i.

To determine the prostate boundary on the TRUS image, the average vector operator is defined:

7 $$C_{i,k} = \arg_{x,y} \max \int {_{\Theta } \int {_{\Omega } } } \phi (x_{out} ) - \phi (x_{in} )dxd\phi$$

where, ${\text{x}} \in \Omega$; $\phi ({\text{x}}) \in \Theta$; ${\text{x}}_{{{\text{in}}}}$ and ${\text{x}}_{{{\text{out}}}}$ are the attraction of the inside and outside of the prostate region to the boundary point, respectively; $\phi$ is a variable range, and $\phi \left( \bullet \right)$ is the magnitude of the attraction within the range. Finding the boundary of the prostate can be expressed as the optimization equation described above. Referring to Hodge^9^, we discretize this expression into an expression that can be iterated continuously over this average vector according to the standard vector operator until the maximum cumulative grey difference is determined. The search description for the ordinary vector diagram is shown in Fig. 7.

The operators for combining semantic constraints are as follows:

8 $$C_{i,k} = \arg \max \left( {\sum\limits_{p = i + 1}^{i + 1} {\phi (IM\left[ {p,k} \right]) - } \sum\limits_{p = i - 1}^{i - 1} {\phi (IM\left[ {p,k} \right])} } \right)$$

**Figure 8:**
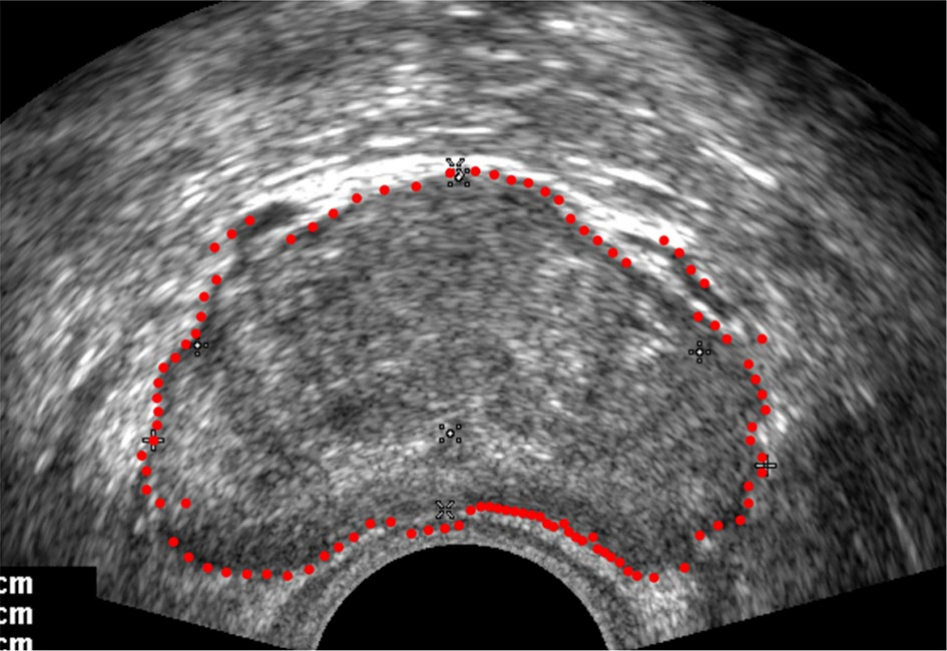
Schematic diagram depicting the preliminary segmentation process (with red used to indicate the resulting segmentation).

Traversing each average vector, the segmentation effect is shown in Fig. 8. The majority of the initial segmentation point set includes the prostate boundary, even though the overall contour is rough and does not achieve a good segmentation effect.

The general outline of the prostate has been isolated, but according to the unique features of the ultrasound image of the prostate. The prostate gland to be segmented should be relatively fixed in position, and its physiological characteristics can make the prostate boundary relatively smooth. The above-described segmentation boundary is rather rough, and there is a situation in which the outliers are partially separated from the boundary. To solve the above problems of rough boundary and outlier points, reduce the distance difference of the point set, and make the segmentation points compatible with the actual boundary points, the neighbourhood information is comprehensively considered, and the original operator C is improved.

**Figure 9:**
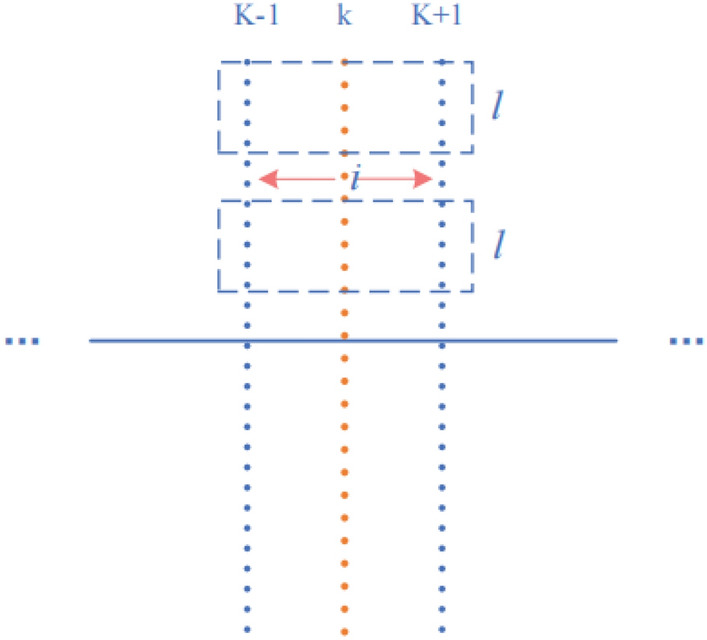
Schematic of the Enhanced Neighbourhood Normal Vector Operator.

Figure 9 shows an improved schematic. Take the point on the Kth normal vector with a row index value $idx_{i,k}$, and its corresponding points on the left and right neighbouring two normal vectors are denoted by idx_i, k−1_ and idx_i, k+1_. Combining the cumulative grey differences of neighbouring normal vectors when calculating the cumulative grey difference of a particular point can reduce the error generated in the search. The segmentation boundary points on the improved Kth normal vector are obtained by the neighbour normal vector operator with the following formula:

9 $$C_{neigh} = \arg \max \sum\limits_{j = k - m}^{j = k + m} {\left( {\sum\limits_{p = i + 1}^{p = i + L} {\phi (IM\left[ {p,k} \right])} - \sum\limits_{p = i - 1}^{p = i - L} {\phi (IM\left[ {p,k} \right])} } \right)}$$

where *m* and m′ represent the number of neighbour normal vectors before and after the Kth normal vector, and *j* represents the range of joint neighbour normal vectors. Compared with *C*, the neighbourhood normal vector operator *C*_neigh_ fully considers the neighbourhood information, while adjusting the value of *m*, which can consider more neighbourhood information.

#### Bidirectional exponential time series denoising

**Figure 10:**
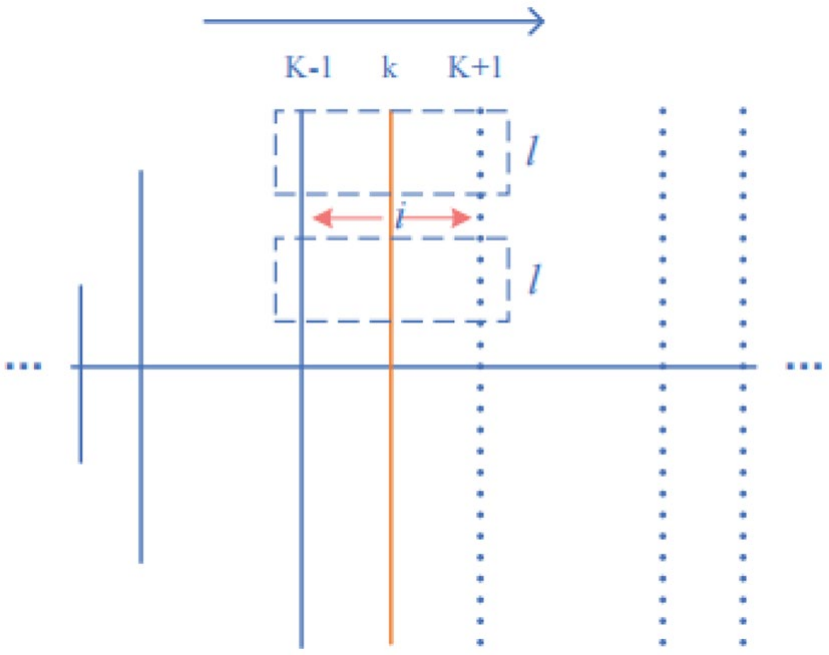
ETSD. weighted NNVO. weights are represented by the length of the normal vector.

After applying the improved neighbourhood average vector operator, it was still found that there was a noise problem in the non-salient region. Firstly, in the balance problem of contour points and noise points, to achieve the purpose of removing the noise, some contour points located in the natural boundary will be distorted and shifted, and the segmentation effect will be reduced. Secondly, the processing of the noise region cannot achieve the ideal segmentation effect. Thirdly, the noise is in the time series because the points in the salient region can be regarded as a collection of center points in continuous time. With the above noise effects, inspired by BiLSTM^22^ to limit the outliers to a specific range and keep the realism of the original contours, we propose a bidirectional hybrid method of exponential time series denoising (ETSD) and improved neighbourhood vector operator (NNVO) in Fig. 10.

With the above-improved operator, the index value where the segmentation point is located is returned $IM\left[ {i,k} \right]$. ETSD gives higher weights to data closer to the time series and lower weights to data farther away from the time series. The lower weighted points have less effect on the current point, making the contour points more accurate.

First, Eq. (10) sets the weights dynamically by iteration rounds to avoid the situation where the algorithm has less data at the initial iteration, and there is a big difference with the original data. Where, $\lambda \in \left( {0,1} \right)$, $(1 - \lambda^{k} )$ to minimize the data bias as the algorithm iterates to a later stage, and iteration rounds *k*.

10 $$\lambda = \min \left\{ {\lambda ,\frac{{1 + {\text{k}}}}{{10 + {\text{k}}}}} \right\}$$

Second, a one-way exponential time series denoising algorithm is defined:

11 $$ETSD_{{\text{k}}}^{(1)} = \left\{ {\begin{array}{*{20}c} {IM\left[ {i,1} \right]} & {k = 1} \\ {\frac{{\lambda \cdot ETSD_{k - 1}^{(1)} + (1 - \lambda )IM\left[ {i,k} \right]}}{{1 - \lambda^{k} }}} & {k > 1} \\ \end{array} } \right.$$

Where $ETSD_{{\text{i}}}$ is the data value after one-way denoising to minimize the deviation of the data from the actual data when the algorithm is iterated to a later stage.

To be more efficient in conjunction with $C_{{{\text{neigh}}}}$, curvature should be introduced to detect anomalous points. The line segment between the neighbouring points and the target point measures the curvature of the target point. The curvature of the target point,$(x_{i} ,y_{i} )$, is defined as follows:

12 $$\theta = \left\{ {{\text{tan}}^{ - 1} \left( {\frac{{y_{i + k} - y_{i} }}{{x_{i + k} - x_{i} }}} \right) - \tan^{ - 1} \left( {\frac{{y_{i} - y_{i - k} }}{{x_{i} - x_{i - k} }}} \right)} \right\}(\bmod 2\Pi )$$

**Figure 11:**
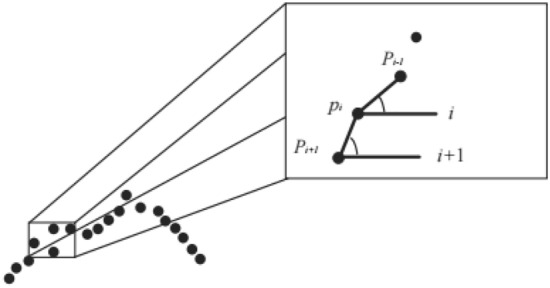
Schematic diagram of outlier detection.

Determine whether $P_{i}$ is an anomaly by considering the spatial relationship of points $P_{i - 1}$,$P_{i}$, $P_{i + 1}$ as shown in Fig. 11. The line formed by $P_{i - 1}$, $P_{i}$ and the line formed by $P_{i}$, $P_{i + 1}$ each form an included angle with the horizontal. With these two included angles $\angle P_{i} P_{i + 1} (i + 1)$ and $\angle P_{i - 1} P_{i} i$, the rate of change of the two line segments can be calculated and reflected in the slope of $P_{i}$. The range of slopes of $P_{i}$ is obtained from the statistical experience of GT images. As a result, a Boolean anomaly detection matrix corresponding to the position $\theta$ can be obtained.

13 $$IM = \left\{ {\begin{array}{*{20}c} {\alpha IM + (1 - \alpha )ETSD} & \theta \\ {(1 - \alpha )IM + \alpha ETSD} & \theta \\ \end{array} } \right.\begin{array}{*{20}c} {is} \\ {is} \\ \end{array} \begin{array}{*{20}c} {normal} \\ {abnormal} \\ \end{array} \begin{array}{*{20}c} {} \\ {} \\ \end{array}$$

Equation (13) combines $C_{{{\text{neigh}}}}$ and a one-way exponential time series denoising. For average boundary points in significant regions, a more considerable weight is set for $IM$ and a smaller weight is set for the $ETSD$; for outliers in non-significant regions detected by (12) a more significant weight is set on the $ETSD$ and pulled back to the average level to obtain the final unidirectional segmentation points.

**Figure 12:**
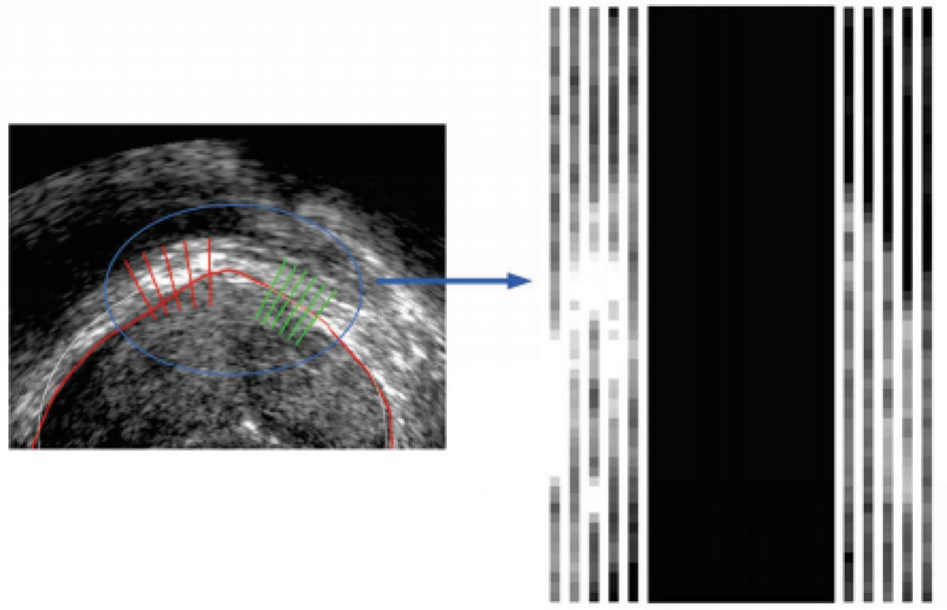
Enlarged schematic diagram of detection area (The region to be detected is indicated in black, flanked by five normal vectors).

For segmentation points located on the actual boundary, these points will deviate from the original actual boundary, resulting in an inaccurate constraint shape. Then, the segmentation accuracy will be reduced. Although we utilize the index-value matrix to eliminate the offset of the data through semantic constraint expression, there is still room for improvement of the segmentation effect in the more common cases, as shown in Fig. 12. Therefore, combining the characteristics of prostate shape data, we utilize the semantic information obtained from the forward input of (13) to push back the data of the current TRUS image in the first round. The equations of the $ETSD$ algorithm for the second round of iterations are as follows:

14 $$ETSD_{{\text{k}}}^{(2)} = \left\{ {\begin{array}{*{20}c} {IM_{N}^{(1)} } & {k = 1} \\ {\frac{{\lambda \cdot ETSD_{k - 1}^{(2)} + (1 - \lambda )IM_{N - K + 1}^{(1)} }}{{1 - \lambda^{k} }}} & {k > 1} \\ \end{array} } \right.$$

Because of noise interference, the unilateral side of the image cannot be accurately segmented by the neighbourhood average vector operator to segment the boundary. When segmenting the middle black region, the algorithmic processing of only one side cannot effectively use the average grayscale and shape features of the other side of the image. In this method, the bidirectional iteration using the grey scale and shape features of the image can make full use of the practical information and obtain better segmentation results. The following Table 1 provides a brief introduction to the algorithm steps of NNVO and ETSD, combined with the above explanation.

**Table 1:**
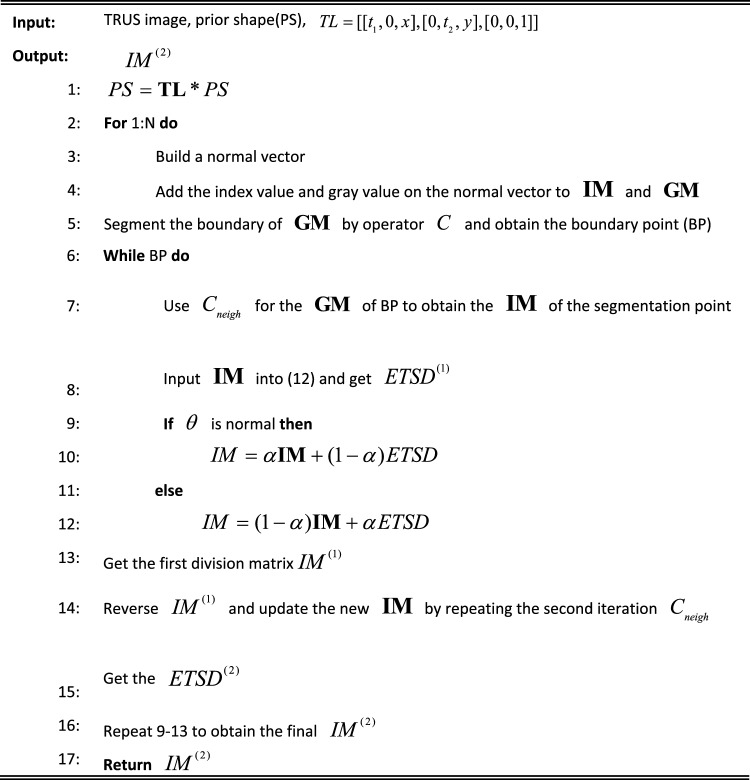
Algorithm of NNVO and ETSD.

From the outset, it was discovered that TRUS images suffer from significant noise. To address this issue, a semantic constraint representation of the cost function is proposed, which leads to improved scoring. However, problematic points exist that are affected by the surrounding environment or by a set of noisy points within a particular region that exhibit robust boundary features, resulting in severe deviations of localized individual points or parts thereof. Moreover, the processing of these points by the adjacent normal vectors in the front and back leads to misinterpretation by the surrounding standard boundary points, resulting in errors in localization. It has been determined from practical studies that the physiological characteristics of the prostate do not support a sudden change in the boundary, mainly when localizing the image of the prostate with a dense set of points, and the set of curved points should be smoothed uniformly. Therefore, it is advisable to take into account the position of a single localized point among all points of the normal vector. This location should not significantly deviate from the relative positions of the initial n localization points in their normal vectors nor the relative positions of the final m normal vectors. Suppose the positioning of the front and back points is simultaneously taken into consideration, along with the relative positions of other segmentation points being given appropriate weighting to relocate the present point. In that case, it may result in better outcomes. By utilizing the pertinent contextual information of the prostate contour in the image, the BiSeC model can effectively tackle the issues of bias and outlier distortion. Furthermore, this approach enhances the precision of segmentation by preserving the genuine boundary's shape characteristics for a significant proportion of high-quality segmentation points.

## Experimentation

### Image acquisition

**Figure 13:**
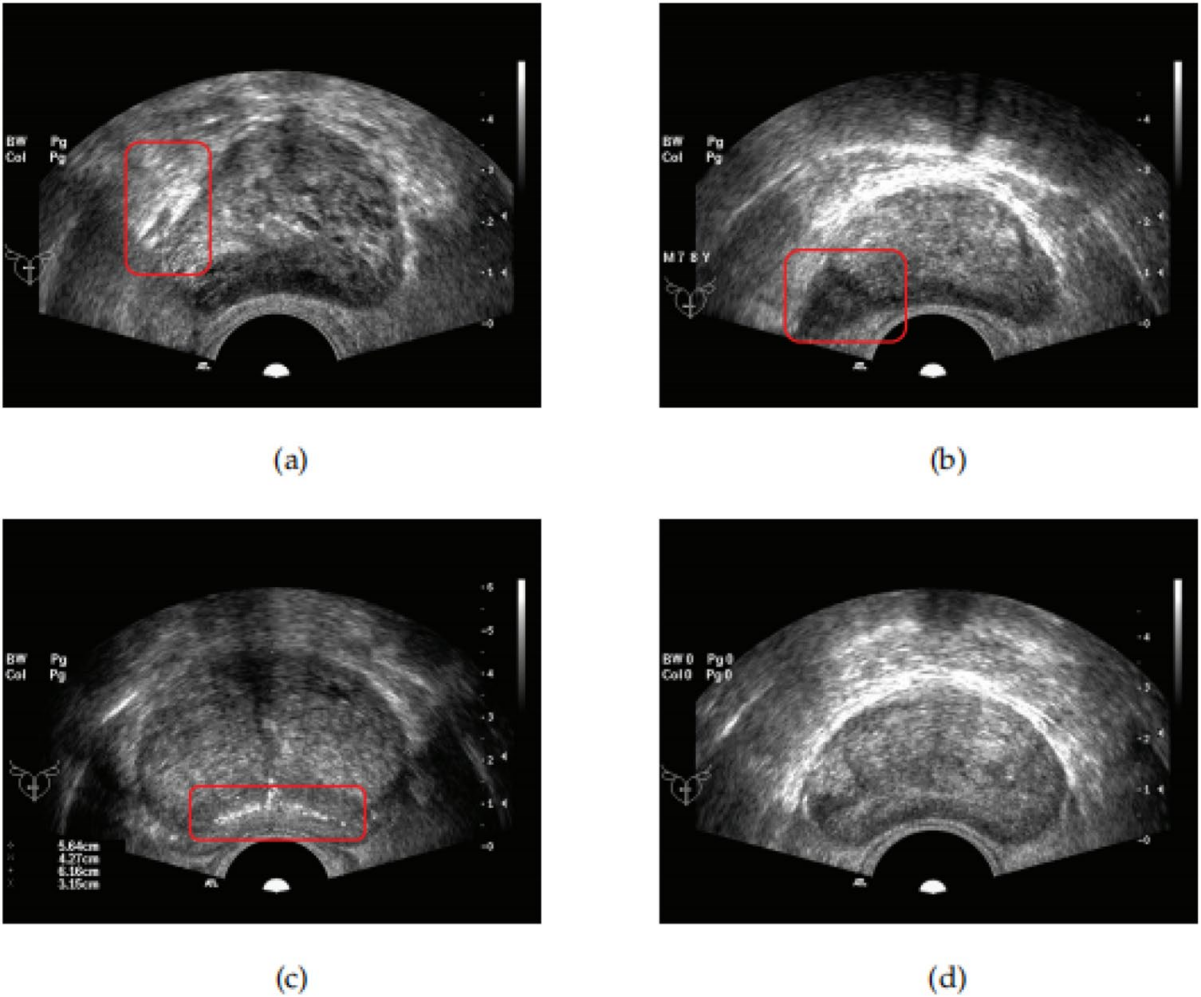
Characteristics of prostate ultrasound images. (**a**) The image is affected by noise, resulting in the loss of boundaries. (**b**) Segmentation is easily affected by artifacts. (**c**) It can easily lead to segmentation errors in the neural network. (**d**) A relatively clear and distinct ultrasound image.

The dataset analyzed in this study was obtained from the open-source database of the Cancer Imaging Archive^23–25^. The US data was collected using Hitachi Hi Vision 5500 7.5 MHz or Nobulus C41V 2–10 MHz end effector probes. The University of California Los Angeles (UCLA) Institutional Review Board approved this study. The open-source database provides information about the ultrasound image connections of a given patient while also referencing available biopsy results. All methods have been confirmed to be carried out by relevant guidelines and regulations, and all experimental plans have been approved. According to reasonable requirements, the data sets used in the current study process are available in The Cancer Imaging Archive^23^ repository, and each human participant agrees to conduct the study of this experiment. For the prostate ultrasound images used in this experiment, the resolution of each image is 768 × 576 pixels, and the measurement value for each pixel is 0.138mm × 0.138mm. Confirm that informed consent has been obtained from all subjects and their legal guardians. Partial images are shown in Fig. 13. The actual boundaries of the prostate in all images are marked by professional doctors practising in the field and then evaluated and corrected by several other doctors. Finally, use it as the fundamental truth value of the target boundary for subsequent segmentation performance evaluation. A total of 132 TRUS data were collected and used in the experiment. In neural networks, the dataset was split into 512 × 512 and extended to use 20% as test data.

### Implementation details

(1) Platform and parameters: The experimental platform relies on PaddlePaddle, which uses the original network without modifications. All networks are set up with dynamic rounds until the model converges. The relevant platform parameters are as follows, Platform: Linux, Python: 3.7.4, GPU: Tesla V100-SXM2-32GB. In the experiments, the parameters are set as follows: Initial weighting factor $\lambda$ = 0.9, N = 100, L = 30,$\alpha$ = 0.9. Network hyper-parameter settings include: Batch_size = 8, LR = 0.01. Momentum = 0.9, Scheduler = PolynomialDecay, Weight_decay = 4.0e-5.

(2) Evaluation metrics: in attempting to evaluate the experimental results obtained in this paper quantitatively, it is decided to perform a comparative analysis in four dimensions: Mean Absolute DDeviation (MAD)^26^, Dice Similarity Coefficient (DSC or Dice)^27^, Intersection on Union (IoU)^28^, and False Positive Rate (FPR)^29^.

15 $$MAD = \frac{1}{{\text{n}}}\sum\limits_{i = 1}^{n} {\left\| {v_{i} - v_{i}^{\prime } } \right\|}$$

16 $$Dice = \frac{2TP}{{2TP + FP + FN}}$$

17 $$IoU = \frac{TP}{{FP + TP + FN}}$$

18 $$FPR = \frac{FP}{{FP + TN}}$$

where v and v′ represent the corresponding points of the image segmentation contour and the truth contour, respectively; TP denotes the overlapping portion of the GT and the resultant image; FN denotes the portion of the GT other than the overlapping portion; and FP denotes the portion of the resultant image other than the overlapping portion.

### Experimentation and analysis

**Figure 14:**
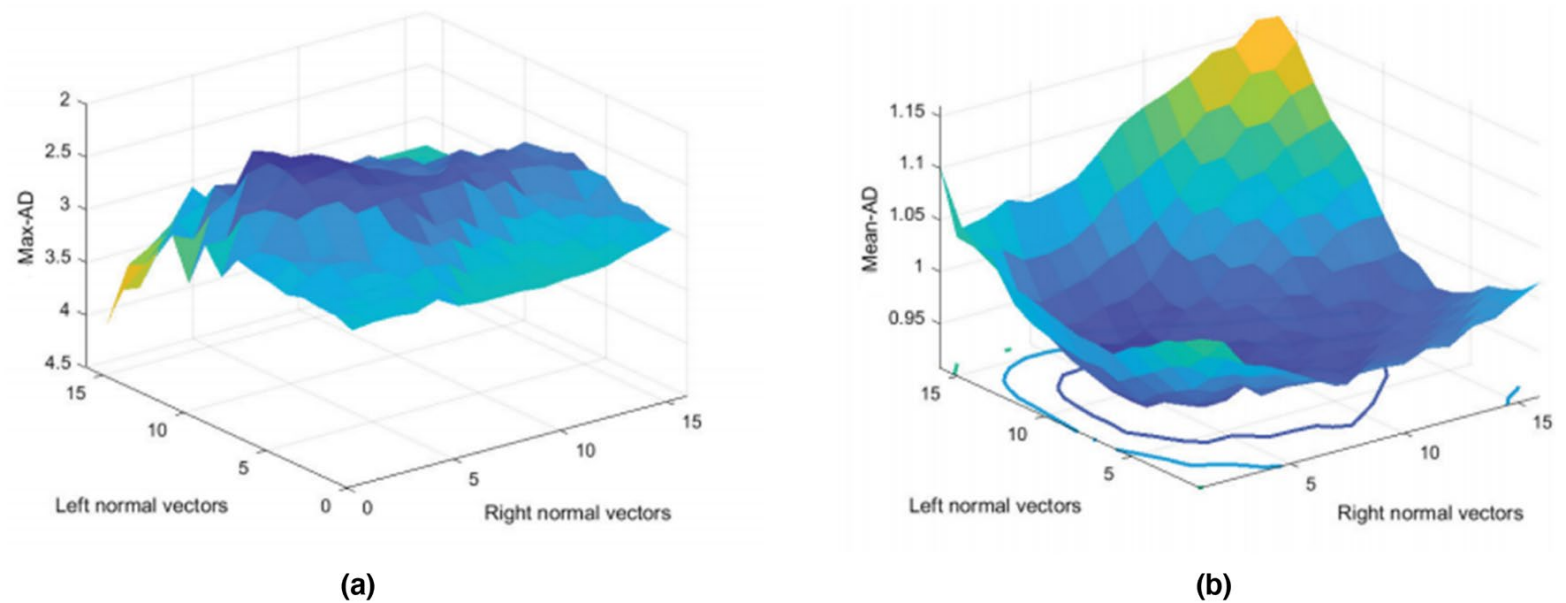
Evaluate the impact of neighbourhood positive vectors on the results. (**a**) showing the maximum absolute deviation for different numbers of left and right normal vectors; (**b**) showing the mean absolute deviation for all experimental images.

To assess the effect of different numbers of neighbourhood normal vectors on the segmentation results, we use different parameters for the comparative study. Avoiding the influence of other algorithms on the segmentation results, this study only explores the segmentation effect of neighbourhood normal vectors, using different numbers of neighbourhood normal vectors and using the Maximum Absolute Deviation (Max-AD) and Mean Absolute Deviation (Mean-AD) for the experiments. The reason for choosing the absolute deviation is that the absolute deviation focuses on reflecting the local deviation. In contrast, the DSC reflects the overall situation, and according to the characteristics of the ultrasound image of the prostate gland, some regions tend to be very difficult, such as the top and bottom sides of the image. In our experiments, we choose numbers in the range of [−15, 15] for discussion. The negative sign indicates the left side, and the results are shown in Fig. 14.

From the above experimental results, Max-AD is most prominent when the left normal vector is taken as 11 and the right normal vector is taken as 4. Mean-AD is smallest when the left normal vector is taken as 10, and the right normal vector is taken as 5. After calculating the errors of [−11, 4] and [−10, 5], we chose [−10, 5] with left normal vector 10 and right normal vector 5 in the subsequent experiments to increase the robustness of the algorithm and realize the end-to-end automatic segmentation.

**Figure 15:**
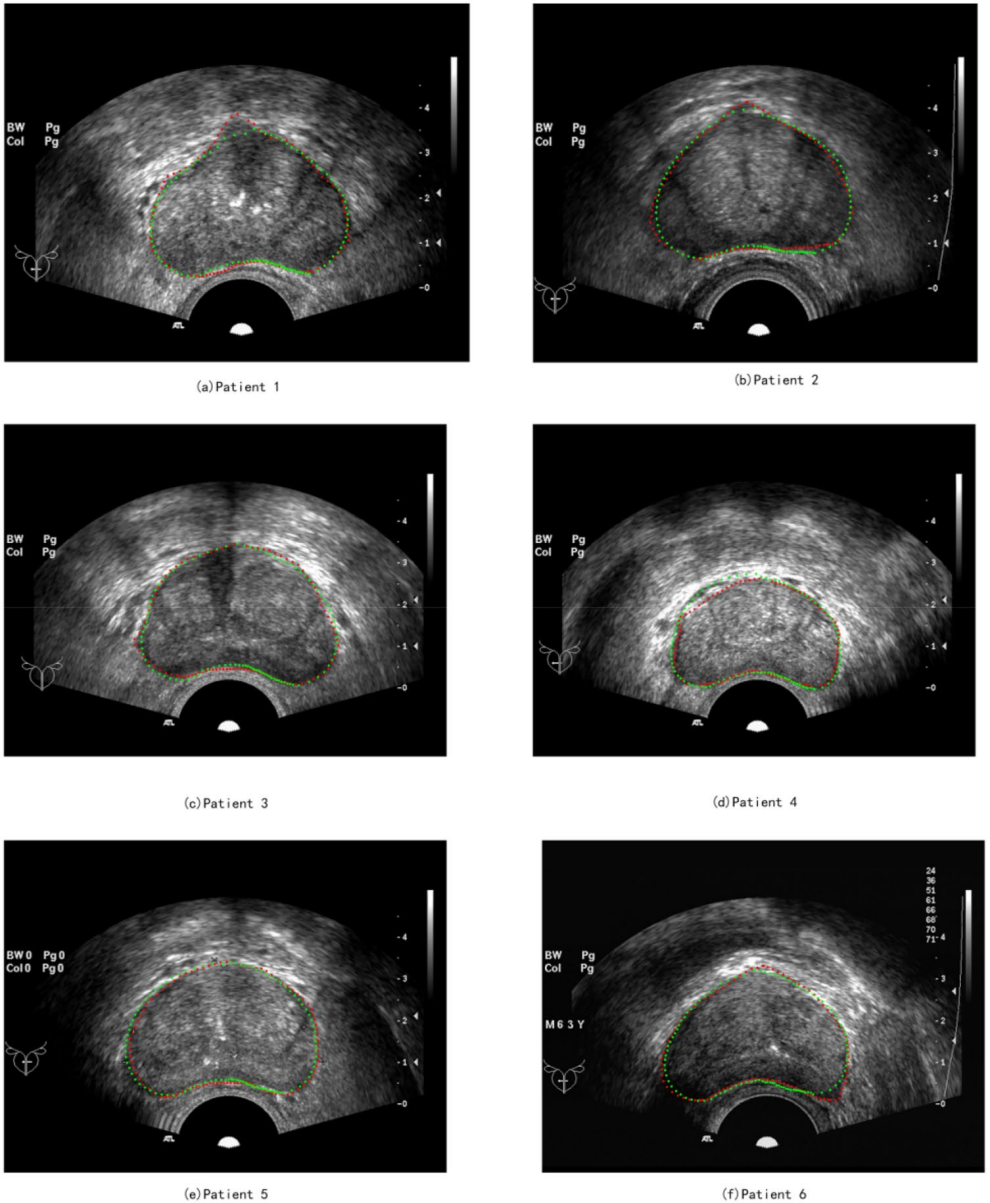
Segmentation results for 6 patients, green for segmentation results, red for ground truth.

**Figure 16:**
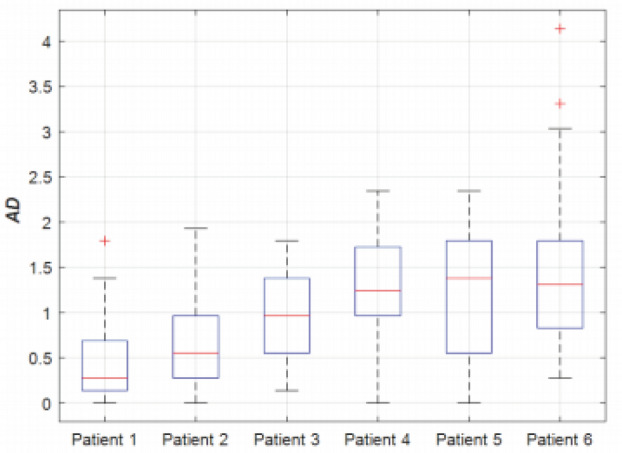
Box plots of mean distances for 6 patients.

Figures 15, 16, and Table 2 provide a visual display and quantitative comparison of segmentation results between different patients. Experimental results show that our method has reached the leading level in the field of prostate ultrasound image segmentation, with a mean value of 96.74% for DSC and 93.71% for IoU. In the face of the drawbacks of pixels such as low resolution, high noise, multiple speckles and multiple artifacts of the image, our method is well adapted to the shape characteristics of different prostates, makes full use of the contextual information, and takes into account the before-and-after trend. For segmented parts that are well segmented and already on the actual boundary, the features can be effectively preserved, and outliers in non-significant regions can be effectively pulled back to normal levels. For example, the DSC of patient 4 is as high as 97.46%, and the IoU is as high as 95.65%. The experimental results also show that our algorithm is susceptible to the part with large curvature edges, which relies to some extent on the accuracy of the average vector. Our algorithm can effectively overcome the pixel defects of ultrasound images and improve the segmentation accuracy.

**Table 2:** Quantitative comparison of segmentation results across patients.

Number	MAD ± STD	Dice (%)	IoU (%)	FPR (%)	Run times (/s)
Patient 1	0.55 ± 0.23	96.36	93.72	1.36	0.34
Patient 2	0.68 ± 0.40	96.22	93.16	3.72	0.35
Patient 3	0.95 ± 0.43	95.26	92.02	8.13	0.37
Patient 4	1.32 ± 0.41	97.46	95.65	1.63	0.42
Patient 5	1.19 ± 0.64	97.06	94.27	3.24	0.35
Patient 6	1.40 ± 0.56	96.13	93.35	2.39	0.41

**Figure 17:**
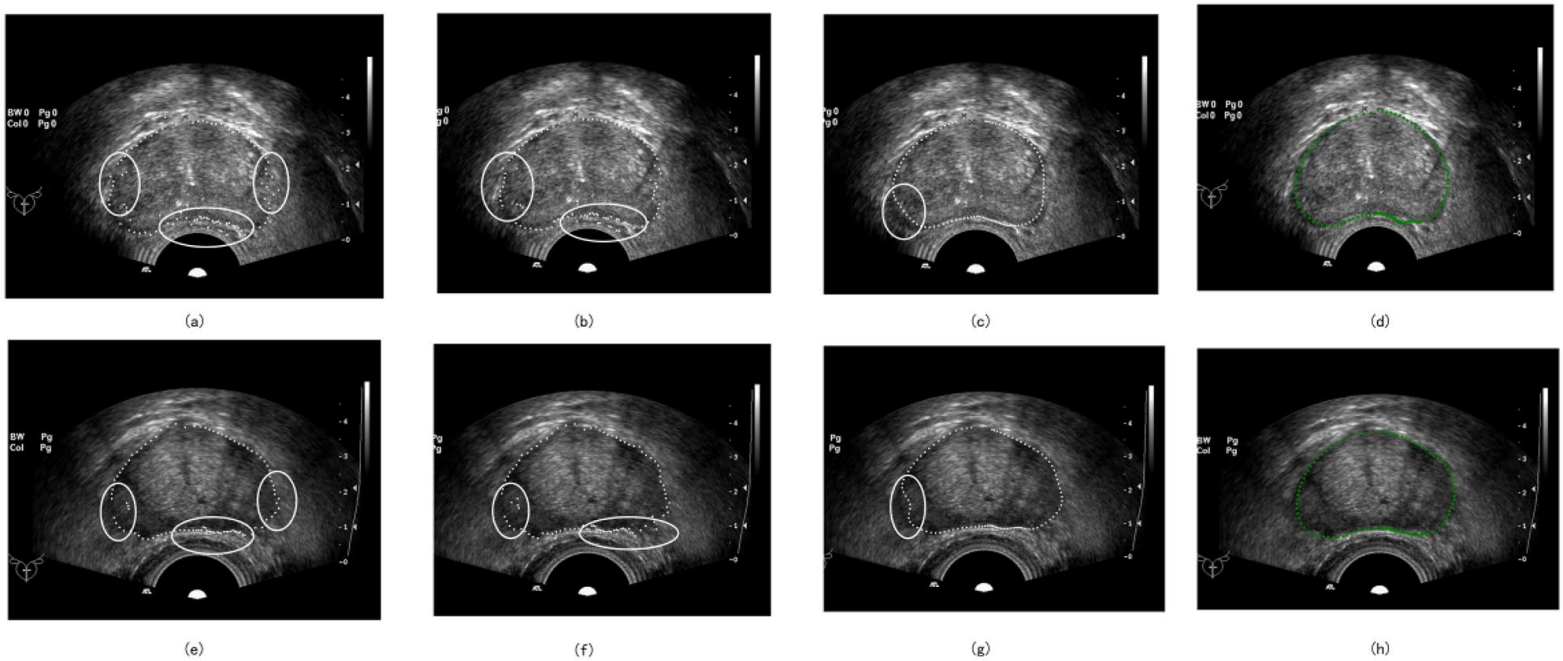
Comparison display of two sets of ablation experiments. (**a**) and (**e**) represent the results obtained with the normal vector operator without combining neighbors; (**b**), (**f**) represent the segmentation results with NNVO only; (**c**), (**g**) represent the results obtained with ETSD leaving the operator; (**d**), (**h**) represent the segmentation results of our method.

The results of the ablation experiments on two TRUS images are shown in Fig. 17, and the MAX-AD results are displayed in Table 3. Hodge's method identifies boundary points at the locations where the cumulative grey difference is the largest, which theoretically should be the actual boundaries of the image. However, due to the influence of a large number of noisy points in the TRUS image, outliers appear in the segmentation results, as shown in (a) and (e). Considering only the neighborhood information, the influence of noise value on the boundary point results cannot be suppressed entirely. This operation causes other errors to some extent, but has little effect on severe outliers, as shown in (b) and (f). Similarly, considering only morphological methods to correct the outliers will seriously deviate from the accurate semantic information, as shown by the labeled regions in (c) and (g). In contrast, our proposed method considers both semantic and accurate pixel information when correcting the outliers. It sets different weights for different degrees of outliers, which achieves good segmentation results, as shown in (d) and (h).

**Table 3:** Max-AD results for two patients.

Indicator	Hodge		Only NNVO		Only ETSD		Ours	
	(a)	(e)	(b)	(f)	(c)	(g)	(d)	(h)
Max-AD	3.17	4.96	3.04	4.55	3.02	5.24	2.89	2.20

**Figure 18:**
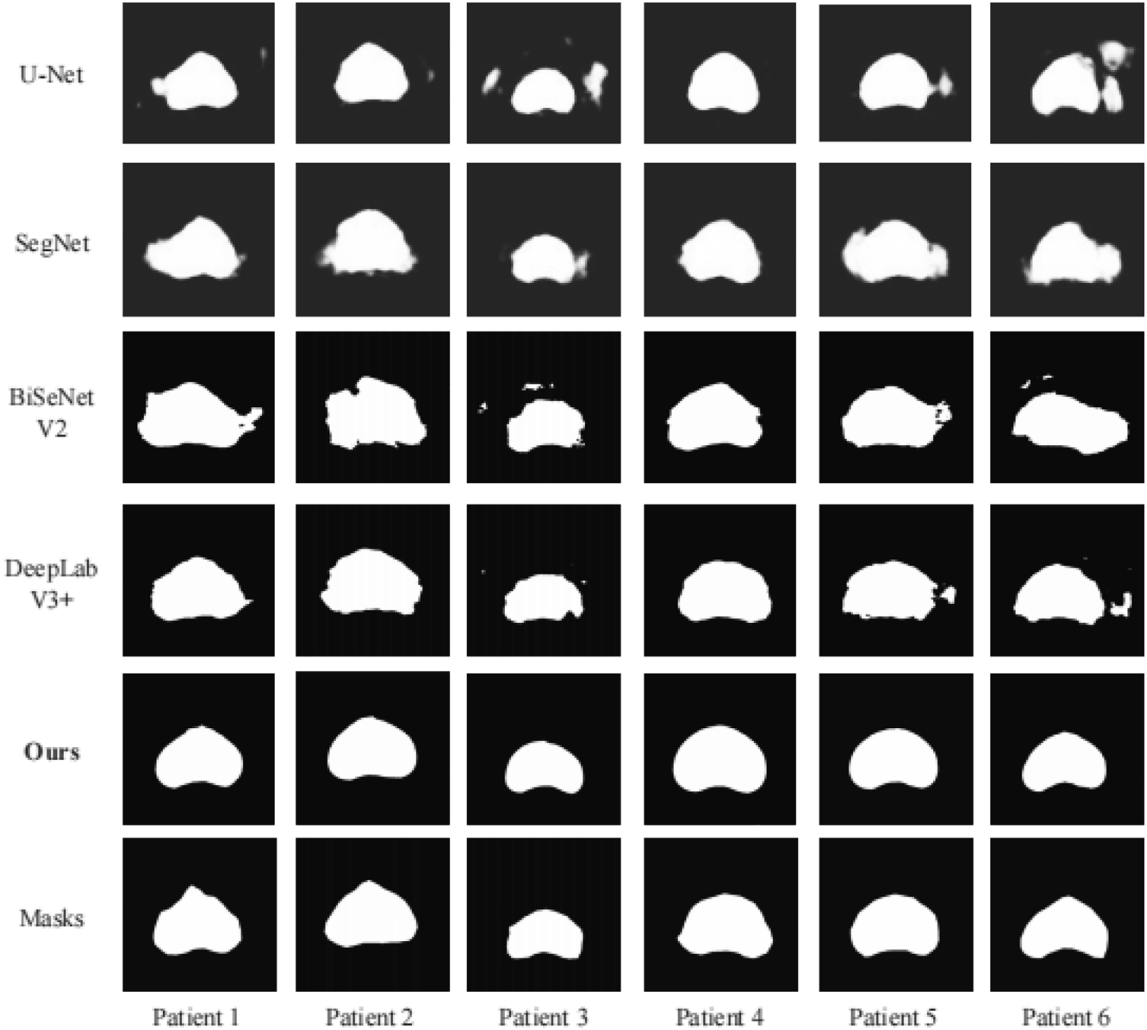
Segmentation results for different methods.

The results above demonstrate that both classical and contemporary networks utilized for prostate ultrasound image segmentation have a shared flaw. When networks suitable for large-scale datasets are applied to small-scale TRUS medical image data, the original image quality and data size cannot meet the requirements of the ideal model. Furthermore, segmentation results indicate that network segmentation methods may produce outliers or blurred boundaries. Figure 18 and Table 4 show the segmentation results of different methods and their quantitative comparisons. For instance, segmentation issues manifested in the results obtained from U-net for Patient 3 and Patient 6 and in the findings from SegNet for Patient 5. Despite the high accuracy level determined by the IoU percentage of 91%, DeepLabV3 + still encountered some challenges. In addition, H-ProSeg^17^ and Bi et al.^30^ are specific methods for prostate segmentation, and the experimental results are shown in Table 4. In particular, for patient 2, segmentation outcomes were skewed by substantial boundary uncertainty, resulting in compromised accuracy. This occurrence is inherent in TRUS images since the neural network operates under a label-oriented mode. We found that the final segmentation data had low DSC and IoU indices due to numerous false positive (FP) index errors present in the network's segmentation results. Although the segmentation results were highly biased, the FP index experienced spikes as a result of the segmented region's outliers, ultimately leading to a sharp decline in segmentation accuracy. However, this paper's proposed method avoids such detrimental circumstances. Our method for segmentation operates on a narrow band created by normal vectors and integrated with shape features to constrain the segmentation effect semantically. By doing so, the issue of region outliers or boundary-blurring, which often occurs in networks, is avoided. Furthermore, our approach offers the benefit of low time consumption while still producing the desired segmentation effect, which is not achievable in neural networks and can ultimately reduce the cost of practical applications.

**Table 4:** Quantitative comparisons of different methods.

Method	Dice (%)	IoU (%)	FPR (%)
U-Net	88.77	80.69	8.31
SegNet	85.84	75.97	8.88
BiSeNetV2	83.87	72.23	11.19
DeepLabV3 +	95.22	91.17	5.92
H-ProSeg	95.80	91.94	5.06
Bi et al.	95.21	–	–
Ours	96.74	93.71	3.99

### Method performance validation

**Figure 19:**
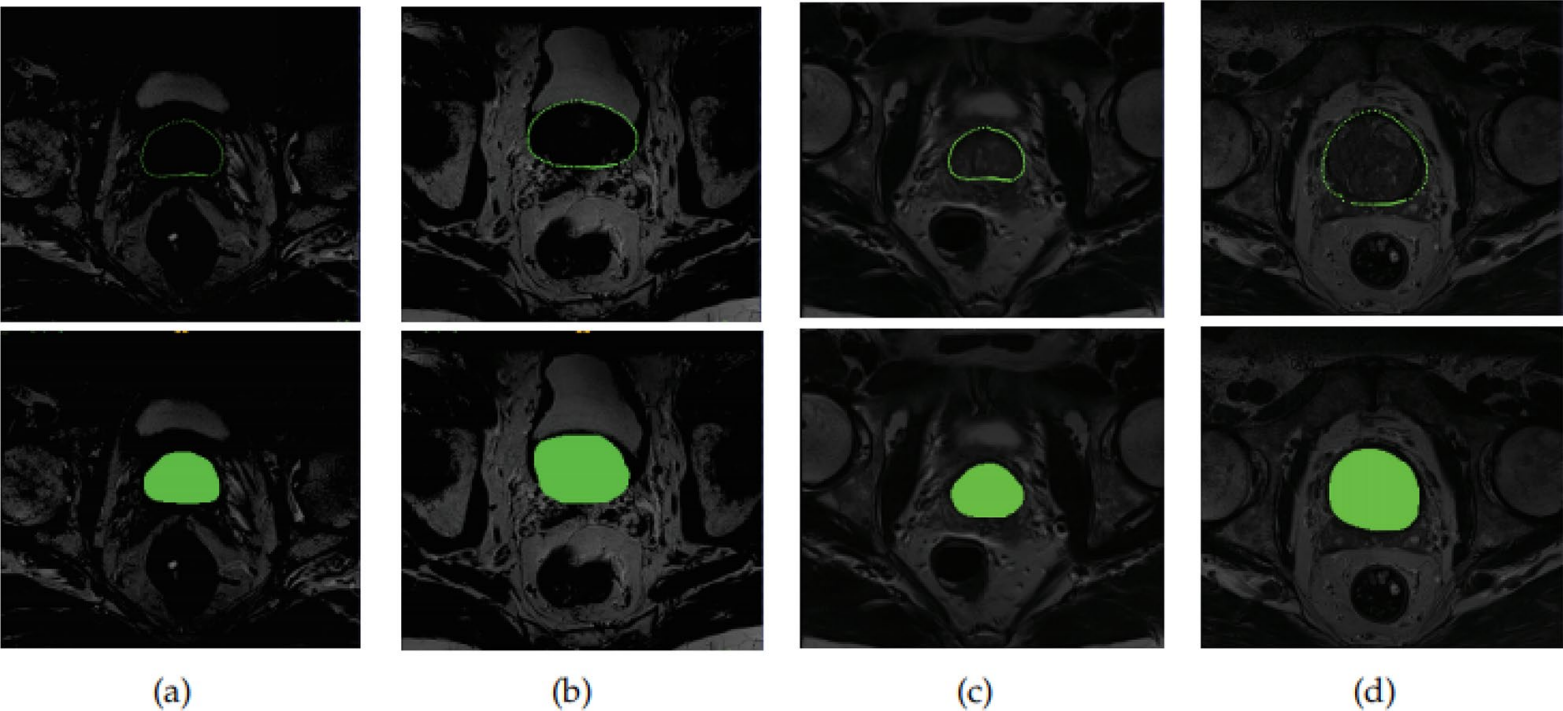
Comparison of four data sets from different slices, with segmentation results in the upper half and labels in the lower half.

We tested the method proposed in this paper on a prostate MRI dataset released by the University Medical Center Nijmegen in the Netherlands^31^. The dataset consists of 48 multiparametric MRI (mpMRI) images of the prostate, including T2-weighted, diffusion-weighted and T1-weighted contrast-enhanced series. We selected 2D slices of a subset of the transverse T2-weighted and Apparent Diffusion Coefficient (ADC) series to validate the method. The validation was complicated by the fact that the prostate shape varied from one 3D slice to another. We tried to use the a priori shape of the same slice to fit the shape of the prostate in different slices, and the following Fig. 19 are the relevant experimental results. Although the priori shape is not very accurate, our method is still able to segment the prostate region efficiently, which suggests that our method is less dependent on the priori shape and localization accuracy, even though it does not train these images, and relies on the feature information of the image itself only, through the model for localization and then using semantic constraints to find the appropriate location, confirming the robustness of the method.

## Discussion

Computerized segmentation of ultrasound images generally achieves average professional accuracy, but obtaining higher levels of segmentation accuracy remains a significant challenge due to the fixed position of the prostate on TRUS images and the theoretically smooth boundary. Compared to other classification tasks, achieving an average level in this task is relatively easy. However, achieving a high level of performance is still challenging due to the large number of noisy points on the TRUS images. Moreover, there is still room for improvement in terms of a single prospective metric. Compounding the difficulty, the number of ultrasound images available for training is severely limited, and the cost of annotation is correspondingly high. Despite collaborations between the National Biomedical Imaging Archive, the Oncology Imaging Archive, and other healthcare organizations^32^ to establish open datasets, the availability of labeled data remains severely limited. For instance, out of the 1500 openly available data in the PI-CAI MRI prostate cancer dataset for 2022^33^, only 220 were labeled data, which is less than 15%, after four institutions (Radboud et al. Center (RUMC), Ziekenhuis Groep Twente (ZGT), University Medical Center Groningen (UMCG) and Norges teknisk-naturvitenskapelige university (NTNU)) jointly reviewed 1500 data. This creates a complicated issue when it comes to existential gradients in label-based deep learning models. Currently, numerous proprietary datasets are available for prostate segmentation^14,34,35^, resulting in a deficient level of error information learned by label-based DL models and limited informative segmentation outcomes.

Our approach avoids the above drawbacks and utilizes the feature information of the image itself for segmentation. Compared to traditional deep learning models, the method is not limited by the size of the dataset and learns error information without the influence of annotations. Despite the use of deep learning models for localization, the algorithm is very tolerant of localization effects and adjusts them through semantically constrained expressions of normal vectors. In terms of accuracy, our method has an average of 96.74% for DSC and 93.71% for IoU. In terms of time consumption, the processing of the index value matrix and grey value matrix reduces the amount of data. It dramatically reduces the time consumption compared to methods that sample the entire image or polar coordinates. Even though the accuracy of this method is inevitably degraded, it can be corrected by subsequent processing. In terms of cost, it does not require significant computational support compared to traditional deep-learning models. The method is less dependent on the previous shape, and the segmentation index decreases when the prostate deviates significantly from the shape.

Although the method proposed in this paper has many advantages compared with most previous studies, we have to admit that there are some shortcomings in practice. In the experiment and analysis section of III, we evaluate the effect of different numbers of neighborhood normal vectors on the segmentation results. This experiment uses all the training data of neural network training. However, the hyperparameters obtained by the experiment are limited by the number of data sets and the amount of noise that can be represented by the data set. This inconsistency in noise distribution is often affected by several factors, such as the doctor's operational proficiency, the disease of the patient's organ itself, and the performance of the ultrasound device. In addition, the patient's prostate organs may have lesions, and the method in this paper is insensitive to huge organ deformation. If the shape of the organ is out of the expression range of PCA, the true boundary of the local organ will be outside the ability of normal vector detection, which may cause wrong segmentation results. At the current level of technology, even complex neural network architectures with strong generalization cannot avoid such errors without training with such data, which will become the focus of our further research.

In future work, we plan to test the robustness of our method on different devices because our prostate images were acquired from the same device. In contrast, the device specifications, resolution, and quality of the TRUS images may differ for different manufacturers. We will also explore the effects of different negative factors and model the effects to improve the segmentation accuracy and optimize the search strategy to improve the segmentation speed due to the presence of noise and artifacts in the TRUS images.

## Conclusion

In summary, this paper aims to address how to obtain more efficient, accurate, and cost-effective TRUS image segmentation, and we propose an end-to-end bidirectional segmentation method for low-resolution, severely noisy and speckle ultrasound images. The method first realizes the automatic localization by the convolutional network, then introduces semantic constraint expression to eliminate the effect of a large amount of noise in the image, and finally combines the neighbourhood normal vector operator and denoising algorithm for bidirectional iteration to achieve the desired segmentation result. Experiments have proved that the method has the advantages of high accuracy, low time consumption, and low cost compared with other algorithms in the field of medical image segmentation.
